# Melatonin: A Dual Protector of Pepper Plants Under Drought Stress via Antioxidant Defence and Glyoxalase-Mediated Cell Detoxification

**DOI:** 10.3390/ijms262211137

**Published:** 2025-11-18

**Authors:** Şebnem Köklü Ardıç, Katarzyna Szafrańska, Ahmet Korkmaz

**Affiliations:** 1 Department of Horticulture, Kahramanmaraş Sütçü Imam University, 46100 Kahramanmaraş, Turkey; 2 Department of Plant Ecophysiology, Faculty of Biology and Environmental Protection, University of Lodz, 90-237 Lodz, Poland

**Keywords:** antioxidant system, *Capsicum annuum* L., drought stress tolerance, glyoxalase mechanism, melatonin, methylglyoxal detoxification

## Abstract

Although the antioxidant role of melatonin in stress mitigation is well established, its multifunctionality may support plant tolerance to drought through additional mechanisms. This study aimed to evaluate melatonin’s contribution to both antioxidant defence and methylglyoxal (MG) detoxification—a harmful compound that disrupts cellular balance under drought stress. The glyoxalase pathway, which is aided by glutathione, plays a pivotal role in MG detoxification. Therefore, we examined the impact of both endogenous and exogenous melatonin on this system. Two pepper genotypes differing in drought tolerance and endogenous melatonin levels were exposed to 12 days of drought following a 5 µM melatonin treatment. The drought-tolerant genotype, characterized by higher levels of endogenous melatonin, exhibited more efficient MG detoxification through increased glutathione and glyoxalase activities, reduced membrane damage and enhanced antioxidant capacity. Exogenous melatonin further mitigated the effects of drought by reducing MG accumulation and stimulating antioxidant and glyoxalase enzymes. Overall, both endogenous and applied melatonin enhances drought tolerance in pepper by activating antioxidant defences and the glyoxalase pathway.

## 1. Introduction

Climate change and global warming profoundly affect water cycles, increasing drought frequency and threatening global food production. With about 64% of the world’s land experiencing drought, agricultural productivity faces serious challenges [1]. Drought restricts plant growth, reducing cell turgor and ultimately impacting crop yield and quality [2].

Drought triggers metabolic adjustments, including aldehyde accumulation, which can be cytotoxic at high levels by modifying proteins and nucleic acids. However, at lower concentrations, aldehydes act as signalling molecules that activate stress responses [3]. Methylglyoxal (MG), a reactive carbonyl species (RCS), exemplifies this dual role. While it activates signalling pathways at low concentrations, excessive MG forms harmful adducts with proteins and nucleic acids, causing cellular dysfunction and ultimately plant death [4,5]. MG is a metabolic byproduct generated through glycolysis, lipid peroxidation, and the oxidative degradation of glucose and glucose-derived proteins [6]. Abiotic stresses such as drought can increase MG levels 2–6 times in plants [7]. Additionally, MG-responsive signalling overlaps with stress-related signalling pathways in plants [8].

**Figure 1:**
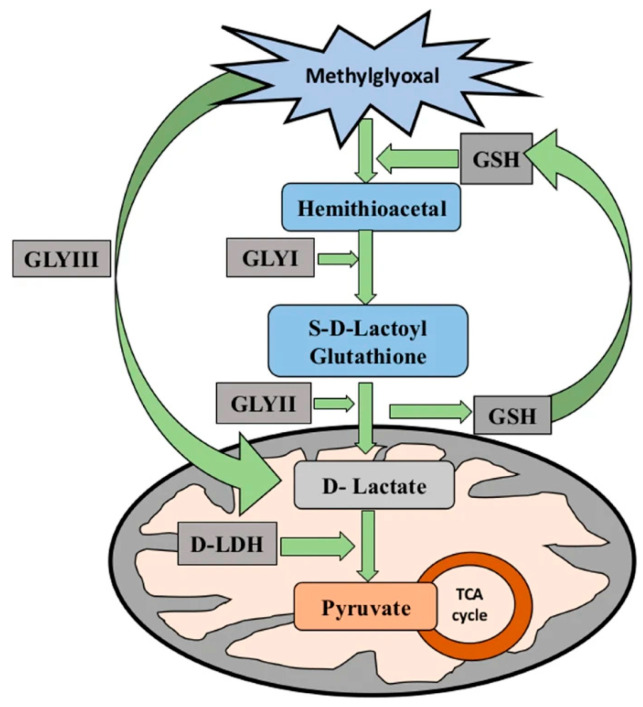
Schematic representation of the methylglyoxal detoxification pathway showing the sequential conversion of methylglyoxal to D-lactate via the glyoxalase system (GLYI, GLYII, and GLYIII) and its further metabolism to pyruvate through D-lactate dehydrogenase (D-LDH). The image is taken from Jain et. al. [9].

The glyoxalase system detoxifies MG, converting it into non-toxic compounds. This system consists of the glutathione (GSH)-dependent enzymes glyoxalase I (Gly I) and glyoxalase II (Gly II), as well as the GSH-independent enzyme, Gly III. The first step in the GSH-dependent pathway involves MG spontaneously reacting with GSH to form a hemi thioacetal. This is then processed by Gly I to produce S-lactoylglutathione (SLG). Next, Gly II hydrolyses the SLG to produce D-lactate, which is then converted into pyruvate by D-lactate dehydrogenase, thus completing the MG detoxification process (Figure 1, [9]). The combined action of Gly I and Gly II is essential for enhancing stress tolerance [10]. Various studies have demonstrated that the GSH-dependent glyoxalase system is upregulated in response to drought stress and that overexpressing the Gly I and Gly II genes can enhance drought tolerance in plants. For example, overexpressing the Gly I and Gly II genes improved the drought resilience of rice and maize by reducing oxidative damage and enhancing antioxidant capacity [11,12]. These findings emphasize the essential role of the glyoxalase system in detoxifying MG in drought-stressed plants, thereby reducing ROS accumulation and mitigating oxidative stress.

In recent years, there has been a growing interest in enhancing the glyoxalase and antioxidant defence systems of stressed plants using natural antioxidants. Melatonin is one such antioxidant biomolecule that stands out as an auxin-like compound with significant protective effects against abiotic stresses such as salinity, drought, and extreme temperatures [13,14,15,16]. GSH, a key antioxidant that maintains redox balance in plant cells, is considered one of the most powerful antioxidants. However, research suggests that melatonin exhibits even greater antioxidant capacity. Due to its strong antioxidant properties, melatonin can alleviate plant stress directly by scavenging ROS or indirectly by inducing antioxidant defences and activating the glyoxalase system [17]. It stimulates the activity of antioxidant enzymes and encourages the production of non-enzymatic antioxidants, thereby mitigating the damage caused by various biotic and abiotic stresses [18]. Exogenous melatonin has been shown to boost the activity of key antioxidant enzymes such as catalase (CAT), peroxidase (POX), superoxide dismutase (SOD), and ascorbate peroxidase (APX) in several crops, including maize (*Zea mays* L.) [19], rapeseed (*Brassica napus* L.) [20], cucumber (*Cucumis sativus* L.) [21], coffee (*Coffea arabica* L.) [22], and naked oat (*Avena nuda* L.) [23]. Additionally, melatonin plays a crucial role in reducing oxidative damage by enhancing the mitochondrial electron transport chain, which lowers free radical formation [24]. Numerous studies have demonstrated that melatonin significantly enhances plant tolerance to abiotic stresses [25]. Its drought-mitigating effects have been repeatedly observed in various crops, including maize (*Z. mays* L.) [26,27,28,29,30], tomato (*Solanum lycopersicum* L.) [31,32,33,34], Tartary buckwheat (*Fagopyrum tataricum* [L.] Gaertn) [35], and cotton (*Gossypium hirsutum* L.) [36]. These findings highlight the potential of melatonin as a sustainable and environmentally friendly way of enhancing agricultural productivity in adverse conditions such as drought.

However, despite extensive research on melatonin’s antioxidant role, its potential involvement in MG detoxification through the glyoxalase pathway remains largely unexplored. Previous studies have rarely examined whether melatonin-induced stress tolerance is linked to the modulation of glyoxalase enzyme activity or GSH metabolism, particularly beyond the germination stage [37,38]. It is essential to understand the underlying mechanisms and adaptive pathways across plant species to optimize melatonin application methods and dosages. Furthermore, studies linking melatonin and the glyoxalase mechanism are scarce, leaving many questions unanswered. Although pepper (*C. annuum* L.) plants are considered one of the most important horticultural crops globally and are known for their response to drought stress, the potential interaction between the glyoxalase defence system and exogenous melatonin treatments has not yet been investigated. In our previous work, we identified a strong correlation between endogenous melatonin levels and drought tolerance among pepper genotypes, suggesting that melatonin content could serve as a physiological marker of stress resilience [39]. Building upon these findings, the present study provides the first integrated analysis of the glyoxalase detoxification system and melatonin signalling in drought-stressed pepper genotypes differing in endogenous melatonin levels. Specifically, we investigate (i) how endogenous melatonin relates to glyoxalase enzyme activity and MG detoxification efficiency under drought, and (ii) whether exogenous melatonin further enhances stress tolerance through the modulation of the glyoxalase pathway.

This approach offers a new perspective on how melatonin improves plant resilience, demonstrating its role in both antioxidant defence and MG detoxification. This is probably achieved by modulating glutathione-dependent glyoxalase activity and maintaining cellular redox balance. Our hypothesis is that melatonin enhances drought tolerance by reinforcing the antioxidant system and stimulating the glyoxalase pathway, which is responsible for methylglyoxal detoxification.

## 2. Results

### 2.1. The Effects of Melatonin on Physiological Parameters Related to Plant Growth Under Drought Stress (Melatonin, Chlorophyll, Proline and Water Potential)

Melatonin content was measured in all experimental variants. In both drought-tolerant and drought-sensitive genotypes, melatonin levels were significantly higher in control plants pre-treated with 5 µM melatonin (CM) and drought-stressed plants pre-treated with 5 µM melatonin (DM) compared with their respective controls (C—control, no stress and D—drought stress). After melatonin application under control conditions, in the drought-sensitive variety, almost a 3-fold increase in melatonin content was observed, while in the tolerant one, this increase was considerably smaller (38%). It is worth noting, however, that under control conditions (C), the initial level of melatonin in the tolerant genotype was almost 5-fold higher than in the sensitive one, and in drought conditions (D), more than twice as high (Table 1).

Chlorophyll content in plant tissues often declines under drought stress, reflecting the impact of water deficiency on photosynthesis. In this study, this process was also observed in the drought-sensitive and drought-tolerant genotypes, although the decrease was slightly higher in the former (28% and 19%, respectively). In both genotypes, however, melatonin contributed to preserving chlorophyll levels during drought stress (40.4 and 63.8 mg kg^−1^ FW, respectively), indicating its protective role (Table 1).

Osmolytes or compatible solutes, such as proline accumulation in plant tissues, are well-known responses to drought stress. Increased proline synthesis under drought stress conditions (D) was observed in both drought-sensitive and drought-tolerant genotypes, although the initial level under control conditions (C) was significantly higher in the latter (24.2 and 54.2 mM kg^−1^ FW, respectively). In both cases, however, the application of exogenous melatonin additionally increased the accumulation of this amino acid (Table 1).

The initial water potential in the tolerant variety was higher than in the sensitive one, both in the control (C) and drought (D) conditions. Melatonin application generally improved water potential under drought conditions in both genotypes, with the tolerant genotype maintaining a higher water potential overall (Table 1).

### 2.2. The Effects of Melatonin on Parameters Indicating Oxidative Damage to Plants Under Drought Stress (H_2_O_2_, Electrical Conductivity (EC), TBARS and Injury Index)

In a drought-sensitive genotype, drought (D) increased the level of H_2_O_2_ (from 1.2 to 1.77 µM kg^−1^ FW), but. melatonin (DM) reduced it to 1.45 µM kg^−1^ FW. In the drought-tolerant genotype, drought stress caused a significant rise in H_2_O_2_ content (from 0.86 to 1.00 µM kg^−1^ FW), but melatonin reduced this further (to 0.91 µM kg^−1^ FW), indicating its role in alleviating oxidative stress (Table 2).

Electrical conductivity (EC) in plant tissues is a key indicator in assessing cellular integrity and stress levels. In the drought-sensitive genotype, drought (D) led to a significant increase in EC (from 75.0 to 88.6%), indicating cell membrane damage due to stress. Under drought conditions, melatonin application (DM) reduced EC to 80.1%, indicating that melatonin mitigated cell membrane damage. In the drought-tolerant genotype, drought (D) increased EC marginally (from 11.6% to 17.8%), although the initial level was almost 7-fold lower than in the sensitive genotype. Under drought conditions, melatonin (DM) lowered EC slightly to 12.2% (Table 2).

TBARS are the by-products of lipid peroxidation and serve as markers of oxidative damage in stressed plant tissues. Significant differences in the level of this marker are visible between the drought-sensitive and drought-tolerant genotypes, even under control conditions (C) (8.7 mM kg^−1^ FW and 5.2 mM kg^−1^ FW, respectively). In the drought-sensitive cultivar, melatonin application did not significantly alter the TBARS content of the control plants. However, in plants exposed to drought stress, it reduced the TBARS content by approximately 20%. Under drought stress (D), the sensitive genotype showed a significantly higher TBARS level (12.4 mM kg^−1^ FW) than the tolerant genotype (6.5 mM kg^−1^ FW), indicating greater lipid peroxidation due to oxidative stress. Although TBARS levels dropped almost to the value recorded under control conditions (C) (5.5 mM kg^−1^ FW), the change in TBARS content in the tolerant genotype was insignificant (Table 2).

**Figure 2:**
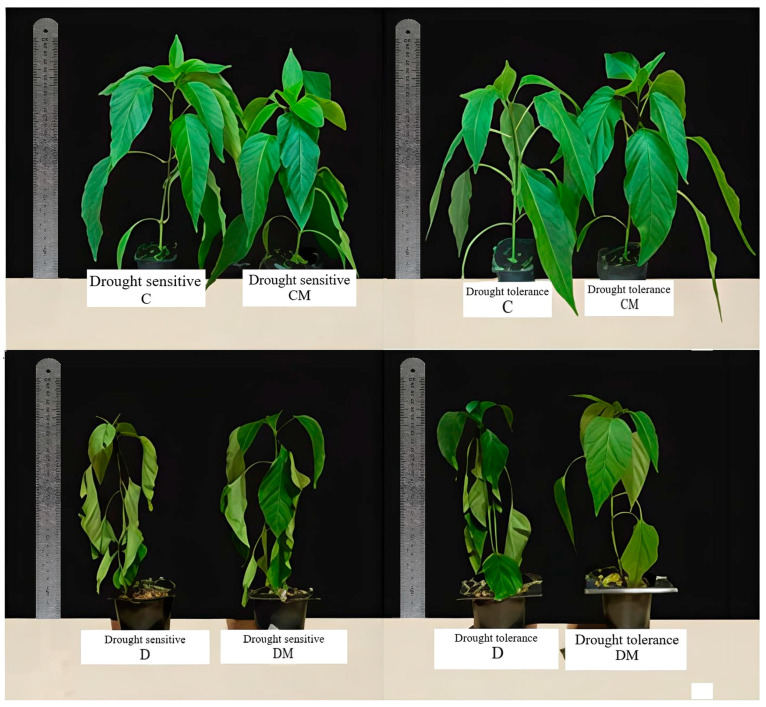
The visual appearance of pepper genotypes that differ in their sensitivity to drought under control and drought stress conditions, with or without 5 µM melatonin pre-treatment (C: control-no stress; CM: control plant pre-treated with 5 µM melatonin; D: drought stress; and DM: drought-stressed plants pre-treated with 5 µM melatonin).

These changes were accompanied by differences in the visible effects of stress damage, as represented by the injury index (Figure 2). Under drought stress (D), this index significantly increased in both genotypes; however, melatonin application mitigated the damaging effects of drought, particularly in the tolerant genotype (Table 2).

### 2.3. Effects of Melatonin Applications on Enzyme Activities

**Figure 3:**
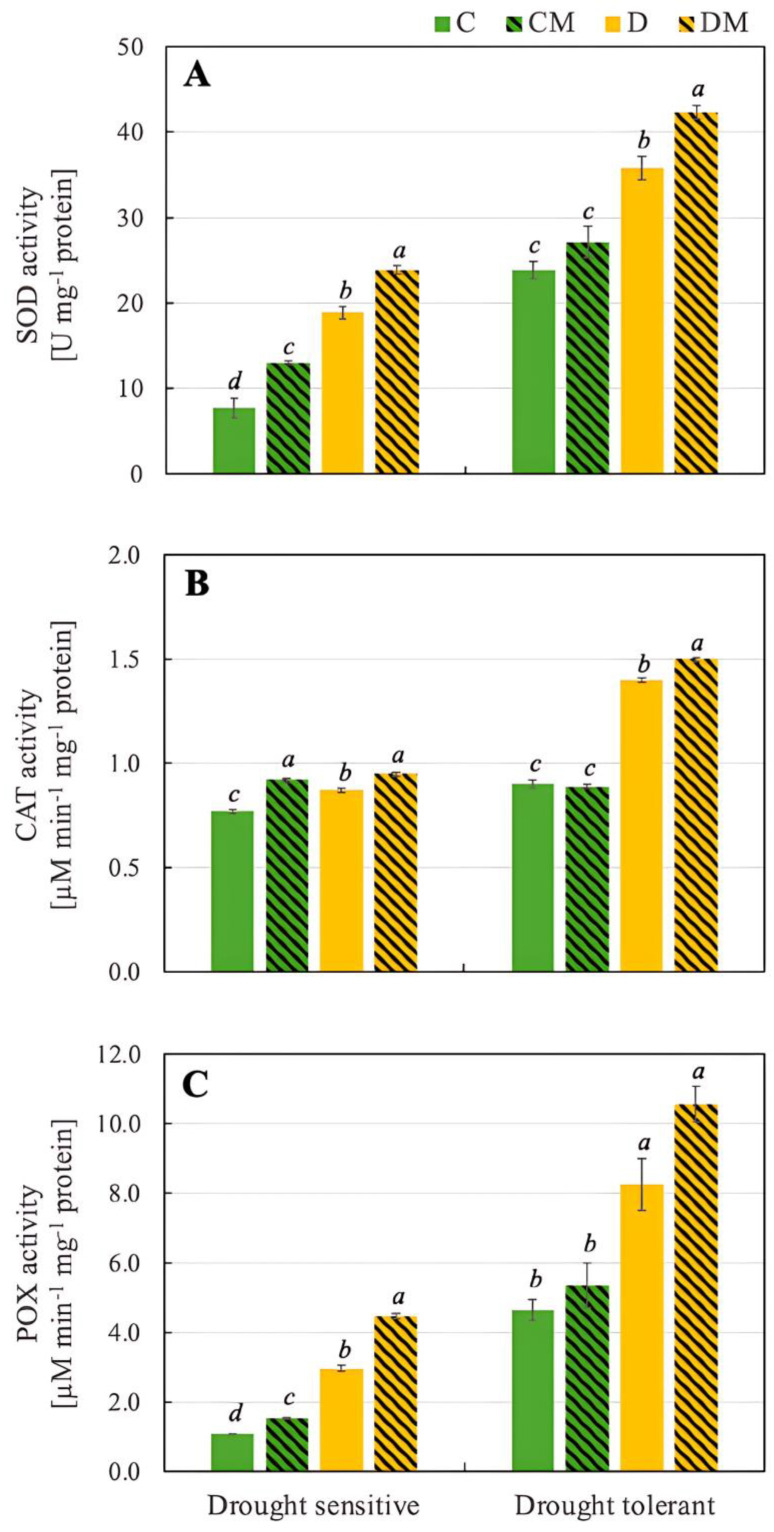
Activity of SOD (**A**), CAT (**B**), and POX (**C**) in pepper seedlings of drought-tolerant and -sensitive genotypes after treatment with melatonin. C: control-no stress; CM: control plant pretreated with 5 µM melatonin; D: drought stress; and DM: drought-stressed plants pre-treated with 5 µM melatonin). Data represent mean values of 4 repeats (*n* = 4) ± SD. Values representing statistically significant differences at the *p* < 0.001 (Tukey’s post hoc test) are marked with lowercase letters.

Antioxidant enzymes such as SOD, CAT and POX play crucial roles in protecting plants from environmental stresses. Both genotypes showed a significant increase in SOD activity under drought stress (D), with the drought-tolerant genotype having a much higher baseline and stress-induced activity (23.9 U mg^−1^ protein and 35.8 U mg^−1^ protein, respectively) than the drought-sensitive one (7.7 U mg^−1^ protein and 18.9 U mg^−1^ protein, respectively). Analyzing the effect of melatonin, it was observed that under control conditions (CM), melatonin modestly increased SOD activity in both genotypes. Under drought stress (DM), however, melatonin further amplified SOD activity (42.3 U mg^−1^ protein), particularly in the drought-tolerant genotype. This indicates that melatonin enhances the plant’s defence against oxidative stress (Figure 3).

Drought stress (D) increased CAT activity in both genotypes, with the drought-tolerant genotype again showing a higher increase (0.87 µM min^−1^ mg^−1^ protein vs. 1.40 µM min^−1^ mg^−1^ protein). Melatonin had a minimal effect under control conditions (CM) in the drought-tolerant genotype, but significantly increased CAT activity in the drought-sensitive genotype. Under drought stress, however, melatonin (DM) led to the highest CAT activity in both genotypes, particularly in the drought-tolerant one (1.5 µM µM min^−1^ mg^−1^ protein) (Figure 3).

In our experiment, drought stress (D) led to a marked increase in POX activity, with the drought-tolerant genotype starting from a more than 4-fold higher baseline and showing a more significant increase than the drought-sensitive genotype (8.26 µM min^−1^ mg^−1^ protein and 2.96 µM min^−1^ mg^−1^ protein, respectively). Under control conditions (CM), melatonin caused a moderate rise in POX activity in the drought-sensitive genotype while maintaining POX activity in the drought-tolerant genotype. Additionally, under drought stress, melatonin (DM) notably increased POX activity, especially in the drought-sensitive genotype (4.48 µM min^−1^ mg^−1^ protein).

**Figure 4:**
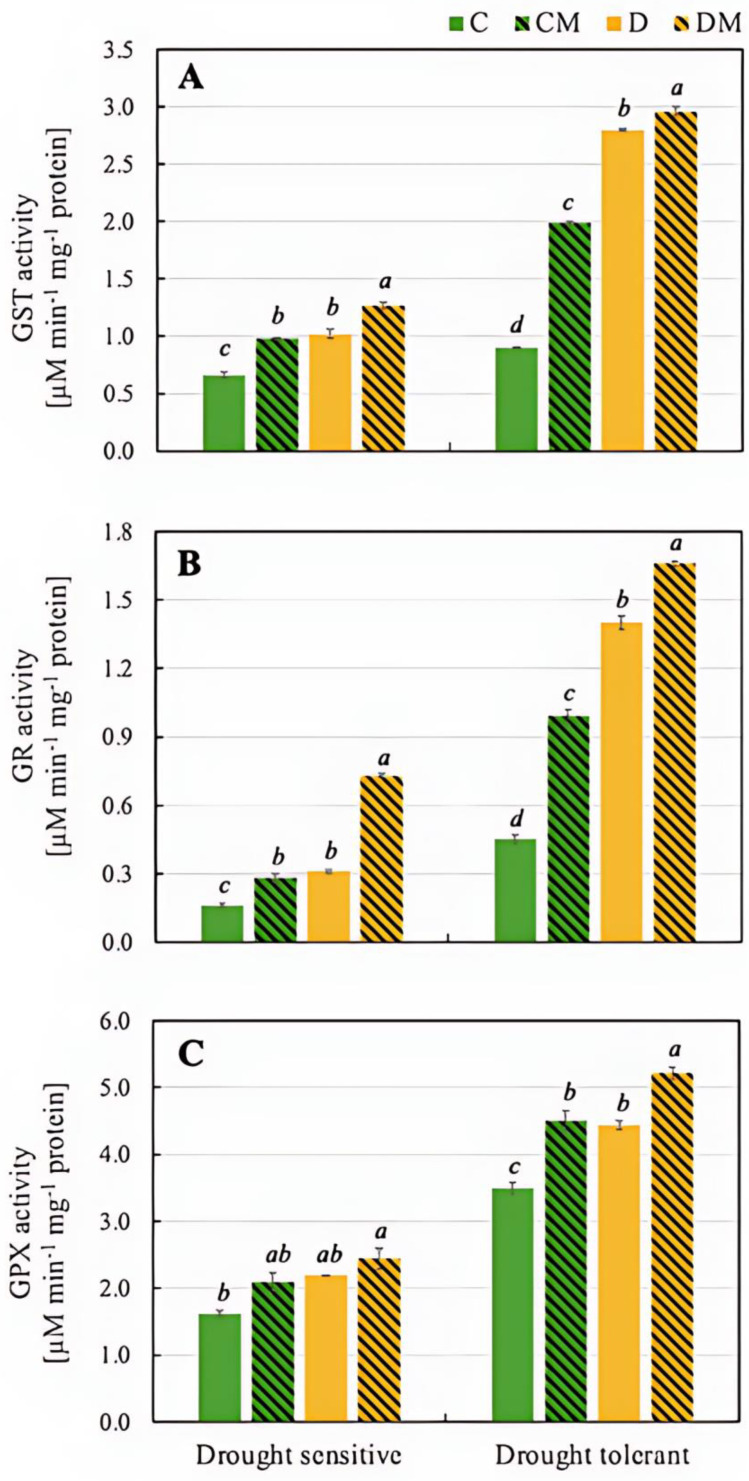
Activity of GST (**A**), GR (**B**), and GPX (**C**) in pepper seedlings of drought-tolerant and -sensitive genotypes after treatment with melatonin. C: control-no stress; CM: control plant pretreated with 5 µM melatonin; D: drought stress; and DM: drought-stressed plants pre-treated with 5 µM melatonin). Data represent mean values of 4 repeats (*n* = 4) ± SD. Values representing statistically significant differences at the *p* < 0.001 (Tukey’s post hoc test) are marked with lowercase letters.

The drought-tolerant genotype exhibited a significantly greater increase in GST activity than the sensitive genotype, particularly under drought stress (D). However, the largest increase occurred when both drought and melatonin were applied (DM) in both genotypes (Figure 4).

The activity of GR increased in response to drought stress (D) in both genotypes, although the basal value was significantly higher in the drought-tolerant one (0.45 µM min^−1^ mg^−1^ protein). Under stress conditions (D), the activity increased in both genotypes, with a twofold increase in the drought-sensitive genotype and a threefold increase in the drought-tolerant one. GR activity also increased in the presence of melatonin (CM), but more so under drought stress conditions (DM), with values exceeding twofold in the drought-tolerant genotype compared to the drought-sensitive one (1.66 µM min^−1^ mg^−1^ protein and 0.73 µ µM min^−1^ mg^−1^ protein, respectively) (Figure 4).

The next enzyme analyzed, GPX, reduces H_2_O_2_ and organic peroxides using glutathione as a substrate. The drought-tolerant genotype exhibited significantly higher GPX activity across all treatments than the drought-sensitive one, achieving the highest activity under DM conditions (5.22 µM min^−1^ mg^−1^ protein) (Figure 4).

### 2.4. Effects of Melatonin Applications on Total Glutathione (GSG), Reduced Glutathione (GSH), Oxidized Glutathione (GSSG), and GSH/GSSG Ratio

The lowest GSG level was recorded in the drought-sensitive genotype under control conditions (C) at 215.4 nM g^−1^ FW. However, melatonin treatment (CM) raised it to 244.9 nM g^−1^ FW. Drought stress (D) caused a significant increase in GSG content to 374.2 nM g^−1^ FW, while melatonin application under drought (DM) further boosted it to 475.5 nM g^−1^ FW. In the drought-tolerant genotype, the baseline GSG level under control conditions (C) was nearly double that of the sensitive genotype (413.8 nM g^−1^ FW), but the response pattern was similar. Drought stress also increased GSG levels, while melatonin application under both optimal (CM) and stressful (DM) conditions further enhanced its accumulation (524.3 and 664.6 nM g^−1^ FW, respectively) (Table 3).

A detailed analysis of the levels of GSH and GSSG revealed that, under control conditions (C) in the drought-sensitive genotype, the level of the antioxidant active form of glutathione (GSH) was very low (20.3 nM g^−1^ FW) in favour of oxidized, non-active glutathione (GSSG) (195.1 nM g^−1^ FW). In contrast, the drought-tolerant genotype exhibited a markedly different response, with GSH levels under control conditions (C) being more than twice as high as GSSG levels. Drought stress (D) led to an increase in GSH content in both genotypes, though the tolerant genotype still accumulated significantly more GSH than the sensitive one. Moreover, melatonin pre-treatment before drought stress (DM) resulted in a significant increase in GSH but a reduction in GSSG contents of both genotypes (Table 3).

These results were reflected in the GSH/GSSG ratio, which indicates the antioxidant status of plant cells. In all experimental combinations involving the drought-sensitive and drought-tolerant genotypes, melatonin increased this ratio. However, under drought conditions, the application of melatonin (DM) to the drought-tolerant genotype undoubtedly induced a rapid, significant antioxidant response, reflected in an extremely high GSH/GSSG ratio of 11.12 (Table 3). Melatonin significantly enhanced the ability of both genotypes to cope with oxidative stress, with more pronounced effects in the drought-tolerant genotype.

### 2.5. The Effects of Melatonin Applications on MG Content and Gly I and Gly II Activities

**Figure 5:**
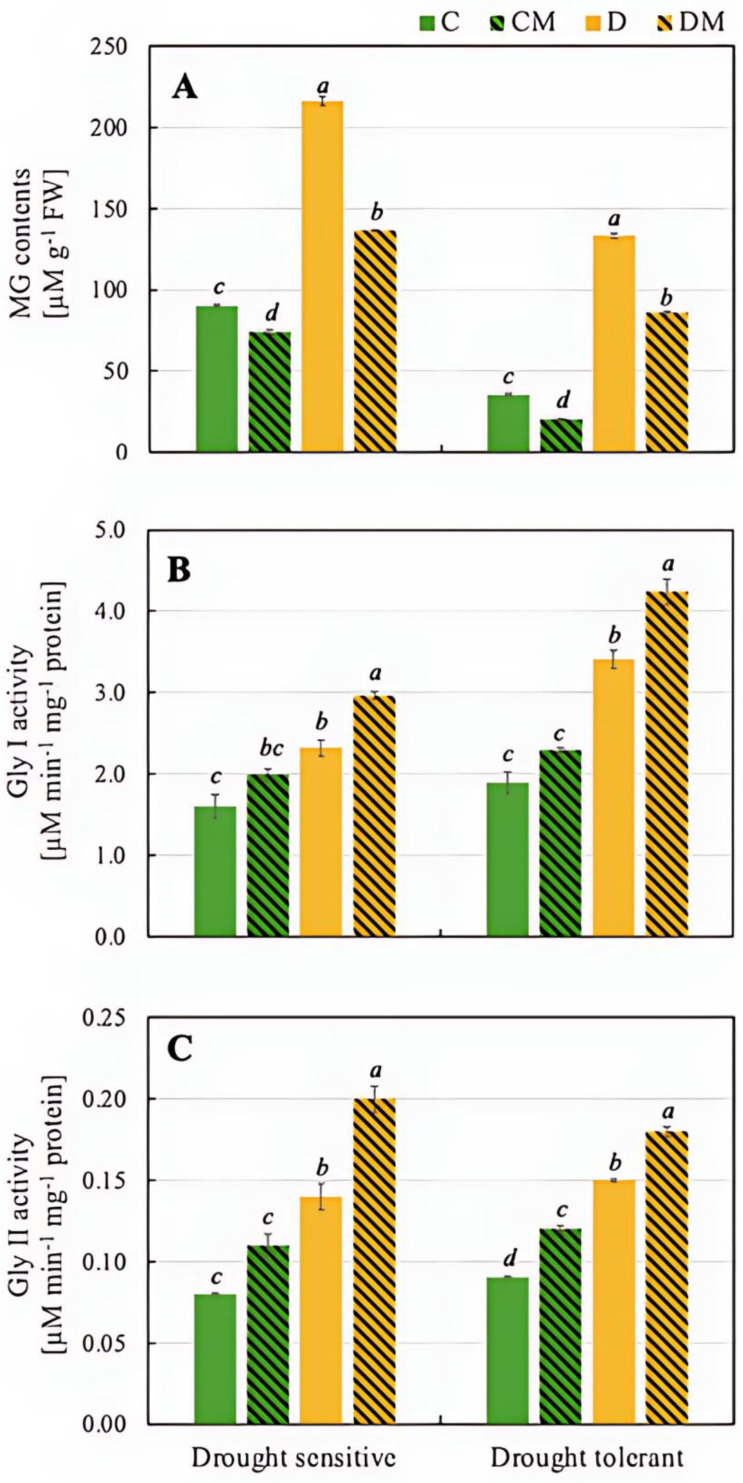
Content of MG (**A**) and activity of Gly I (**B**) and Gly II (**C**) in pepper seedling of drought-tolerant and -sensitive genotypes after treatment with melatonin. C: control-no stress; CM: control plant pre-treated with 5 µM melatonin; D: drought stress; and DM: drought-stressed plants pre-treated with 5 µM melatonin). Data represent mean values of 4 repeats (*n* = 4) ± SD. Values representing statistically significant differences at the *p* < 0.001 (Tukey’s post hoc test) are marked with lowercase letters.

In the drought-sensitive genotype, drought stress (D) led to a significant increase in MG content (216.0 µM) compared to the control (C) (89.9 µM), reflecting elevated stress levels. Under drought stress with melatonin treatment (DM), MG levels decreased to 136.2 µM, demonstrating melatonin’s ability to reduce MG accumulation under stress. Similarly, in the drought-tolerant genotype, MG content increased from 35.2 µM in the control (C) to 133.4 µM under drought stress (D). However, melatonin under drought (DM) reduced MG to 86.0 µM, showing that melatonin also lowers MG in the tolerant genotype. In both genotypes, melatonin significantly reduced the accumulation of toxic MG under drought conditions, with a somewhat stronger effect observed in the drought-tolerant genotype (35.5% reduction vs. 37.0%) (Figure 5).

Analysis of Gly I activity revealed an increase in the drought-sensitive genotype under drought stress (D), rising from 1.60 µM min^−1^ in the control (C) to 2.32 µM min^−1^, indicating activation of detoxification mechanisms. Melatonin treatment (DM) further increased Gly I activity to 2.96 µM min^−1^, demonstrating its role in enhancing the plant’s detoxification capacity. In the drought-tolerant genotype, Gly I activity was higher still under drought stress (D), reaching 3.41 µM min^−1^ compared to 1.89 µM min^−1^ in the control (C). Melatonin application under drought (DM) boosted activity further to 4.24 µM min^−1^ (Figure 5).

Changes in the profile of Gly II activity were very similar in both drought-sensitive and drought-tolerant genotypes. In the former, Gly II activity increased under drought conditions (D), reaching 0.14 µM min^−1^ compared to 0.08 µM min^−1^ in the control (C) group. When melatonin (DM) was applied, Gly II activity increased further to 0.20 µM min^−1^, indicating that melatonin effectively boosts Gly II activity under drought stress. In the drought-tolerant genotype, drought stress (D) led to an increase in Gly II activity compared to the control (C) (0.15 µM min^−1^ and 0.09 µM min^−1^, respectively). The application of melatonin under drought conditions (DM), triggered a further increase in Gly II activity to 0.18 µM min^−1^ (Figure 5).

## 3. Discussion

Drought is one of the most severe abiotic stresses limiting pepper (*C. annuum* L.) productivity worldwide, primarily by disrupting water balance and inducing oxidative and carbonyl stress. In this study, melatonin emerged as a key regulator mitigating these adverse effects. Comparative analysis of drought-tolerant and drought-sensitive genotypes revealed that plants with higher endogenous melatonin levels exhibited greater drought resilience, which was further enhanced by exogenous melatonin treatment.

These findings highlight that melatonin contributes to stress tolerance not only through its well-established antioxidant activity but also by stimulating methylglyoxal detoxification via activation of the glyoxalase pathway. This dual regulatory role provides a broader understanding of melatonin’s function in maintaining cellular homeostasis under drought conditions. Abundant evidence suggests that drought-induced accumulation of endogenous melatonin plays a key role in increasing plant stress tolerance by eliminating ROS. This effect has been observed in many crop plants as rice (*Oryza sativa* L.) [40], wheat (*Triticum aestivum* L.) [14], barley (*Hordeum vulgare* L.) [41], apple (*Malus domestica* Borkh.) [42], grape (*Vitis amurensis*, *V. vinifera* and *V. labruscana*) [43], and pepper (*C. annuum* L.) [39]. Melatonin pre-treatment has been shown to induce overexpression or transient expression of melatonin biosynthetic genes, thereby increasing endogenous melatonin levels and improving plant stress tolerance [39]. Consistent with these findings, our study demonstrated a significant increase in endogenous melatonin content following exogenous application, particularly under drought stress. While a threefold increase was observed in the sensitive genotype under control conditions, this rate remained at 38% in the tolerant genotype. Initial levels were higher in the tolerant genotype under all conditions (Table 1). This suggests that exogenous melatonin can additionally stimulate melatonin biosynthesis pathways, contributing to improved stress resistance. Similarly, previous research also reported upregulation of melatonin biosynthesis genes following melatonin treatment [44,45,46,47,48,49,50].

Photosynthesis is the main process that enables plants to grow and develop properly, so maintaining high chlorophyll content and ensuring efficient functioning under stressful conditions is extremely important. Although drought reduced chlorophyll levels in both the sensitive and tolerant genotypes, it is worth noting that it was more than twice as high in the drought-tolerant genotype. In both cases, melatonin application further increased the content of this photosynthetic pigment (Table 1). This suggests that melatonin significantly delays leaf senescence and stabilizes photosynthetic functions. Similar results were obtained in cotton [51] and tomato [44], confirming the role of melatonin in reducing chloroplast damage and downregulating the expression of many crucial genes in chlorophyll degradation. As demonstrated by Szafrańska et al. [52], melatonin may help to preserve chlorophyll content by stimulating the production of metabolites involved in its biosynthesis and slowing down its breakdown.

In addition to ensuring the efficient functioning of the photosynthetic apparatus, melatonin influences the accumulation of proline in drought-stressed plants, playing an extremely important osmoprotective role. In the pepper plants that we studied, drought stress increased proline accumulation in both the drought-sensitive and the drought-tolerant varieties. However, it is important to note that the initial level was higher in the tolerant one. Following the application of exogenous melatonin, proline levels increased further in both genotypes, thereby promoting drought tolerance mechanisms (Table 1). Similar results were obtained by Mushtaq et al. [53] in Micro-Tom tomato plants treated with melatonin, where, in addition to proline, the levels of other osmoprotectants (trehalose, fructose, sucrose, starch, and glycine betaine) also increased significantly under drought stress. Additionally, the application of melatonin increased the expression of genes encoding osmoprotectants such as SlP5CR, SlP5CS, SlSPS, SlBADH, SlT6PS, and SlSUS3. This confirms that melatonin participates in the enhancement of osmotic balance and stress tolerance. Increased levels of proline and other osmolytes after melatonin treatment under drought were also reported by Kaya and Shabala [54] in pepper plants, Eisa et al. [55] in ‘butter cup’ (*Ranunculus asiaticus*) and El Yazied et al. [56] in potato (*Solanum tuberosum* L.). These results suggest that melatonin can enhance the production of osmotic solutes, helping to reduce water loss and stabilize the cell membrane during drought stress. The water potential of the analyzed pepper plants also confirms the fact that melatonin favourably affects plant water management under drought conditions. The tolerant genotype had a higher water potential under both optimum and drought conditions, and melatonin treatment improved water potential in both genotypes (Table 1). These findings highlight the potential of melatonin as a valuable tool for enhancing plant resilience to drought stress in the context of climate change.

Unfortunately, stressful conditions such as drought greatly affect the redox status of cells, resulting in oxidative damage caused by the accumulation of ROS. This study highlights the impact of drought stress on cellular integrity and oxidative stress in both drought-sensitive and drought-tolerant genotypes. Drought induced an increase in H_2_O_2_ content, resulting in cell membrane damage, as evidenced by elevated EC and TBARS levels, particularly in the susceptible variety. However, melatonin was found to mitigate this damage to some extent. Furthermore, melatonin treatment reduced drought damage by decreasing the injury index, particularly in the tolerant genotype (Table 2). These findings are supported by recent studies, including those by Talaat [57] and Yang et al. [51], which highlight melatonin’s role in enhancing drought tolerance by improving oxidative stress management, membrane stability, and overall physiological function.

In addition to the effects described above, melatonin also strengthens the plant defence system by increasing the activity of antioxidant enzymes. These enzymes play a crucial role in mitigating oxidative stress caused by drought, helping plants maintain cellular homeostasis and survive adverse environmental conditions. The increased activities of SOD, CAT, and POX noticed in this work (Figure 3) are consistent with studies of Kaya and Shabala [54] and El-Yazied et al. [56], showing that melatonin enhances the antioxidant defence system. The more pronounced response in the drought-tolerant genotype, with higher endogenous melatonin level, suggests that exogenous melatonin treatment boosts the already strong antioxidant machinery, making the plants more resilient to oxidative stress. These enzymes play crucial roles in scavenging ROS, thereby protecting the plants from the damaging effects of drought-induced oxidative stress. This is indicated by the reduced levels of H_2_O_2_, TBARS, and EC in the studied pepper plants (Table 2). In the study by Mushtaq et al. [53] on tomato plants subjected to drought stress, in addition to an increase in the activity of CAT, SOD, APX, DHAR, POD, and GR induced by melatonin, overexpression of related antioxidant genes (SlCAT1, SlAPX, SlGR, SlDHAR1, SlPOD, and SOD) was also observed. This suggests melatonin has great potential to enhance plant stress resistance in a changing climate, already at the stage of gene expression.

Building on these findings, analysis of all stress markers discussed above highlights the importance of endogenous melatonin levels in pepper plant tissues as a key factor in determining their response to drought stress. The application of exogenous melatonin further amplifies this stress tolerance. To gain a deeper insight into melatonin’s role in drought tolerance, the following section will focus on its potential impact on glutathione reserves and the regulation of the glyoxalase pathway, as current data on these areas is limited. The glyoxalase pathway, tightly linked to the glutathione system, plays a pivotal role in detoxifying MG, a harmful by-product that accumulates under stress conditions. GSH is a major antioxidant that helps maintain cellular redox balance by directly scavenging ROS and participating in enzymatic detoxification processes [58]. Therefore, its level in cells is extremely important for this system to be effective. Melatonin enhanced the synthesis and recycling of GSH through the activation of key enzymes such as GR, which regenerates reduced GSH from its oxidized form (GSSG), and GPX, which uses GSH to reduce H_2_O_2_ and lipid peroxides, limiting oxidative damage (Figure 4). Additionally, GST plays a role in detoxifying harmful compounds by conjugating them with GSH, a process that melatonin supports through its regulatory effects. After melatonin treatment, Cui et al. [14] noted an increase in the activity of antioxidant enzymes associated with glutathione (GST, GR, and GPX) in wheat seedlings, and Bidabadi et al. [59] reported the increased GR activity in two *Salvia* species under drought stress. In the current study, both pepper genotypes showed elevated activity of these enzymes, with a notably stronger response in the drought-tolerant genotype (Figure 4).

When plants encounter stressful conditions such as drought, elevated levels of GSSG typically lead to a reduced GSH/GSSG ratio. However, in our study, melatonin application under drought stress resulted in elevated GSH levels and decreased GSSG levels, thereby boosting the GSH/GSSG ratio (Table 3). This aligns with findings by Hasanuzzaman and Fujita [60] in rapeseed (*B. napus*) seedlings and Wang et al. [42] in apple (*Malus domestica* Borkh) leaves, where melatonin enhanced the GSH pool and reinforced the significance of the GSH/GSSG ratio as an oxidative stress marker. Similarly, Galano et al. [61] demonstrated that foliar melatonin application in wheat plants under stress conditions elevated total GSH levels and improved the GSH/GSSG ratio compared to controls, while Bidabadi et al. [59] reported comparable results in melatonin-treated *Salvia* plants.

Beyond its role in amplifying GSH levels and related enzyme activities, melatonin also regulates other critical defence systems, such as the glyoxalase pathway. This aspect is particularly interesting, given how little data there is on the role of melatonin in regulating this cycle. For instance, in maize seedlings subjected to heat stress, melatonin activated enzymes in the MG detoxification system, specifically Gly I and Gly II [15]. Similarly, in tomato seedlings under salinity stress, melatonin application combined with gibberellic acid reduced MG content by enhancing the activity of these enzymes [62]. The dual role of melatonin in managing oxidative stress and detoxifying MG further highlights its versatility as a key regulator of stress tolerance in plants. The current study provides further evidence of this regulatory role in pepper plants, where the sensitive genotype had significantly higher MG content than the tolerant one. This finding underscores the influence of endogenous melatonin levels on the accumulation of this reactive carbon species. Notably, exogenous melatonin in both genotypes increased the activity of Gly I and Gly II, leading to a marked reduction in MG levels in plant tissues (Figure 5). These results suggest that while lower endogenous melatonin in the sensitive genotype may contribute to less efficient MG detoxification, exogenous melatonin can compensate by activating the glyoxalase enzymes. This increased enzymatic activity reduces MG accumulation and mitigates oxidative stress, protecting cellular integrity.

The important role of the glyoxalase cycle in response to abiotic stresses was confirmed by the latest research by Arman et al. [63]. An in silico analysis of *C. annuum* identified 19 CaGLYI, 9 CaGLYII, 3 GLYIII (CaDJ-1), and 11 D-LDH members of the glyoxalase and D-lactate dehydrogenase gene families. Expression profiling revealed variable responses under stress, with upregulation of CaGLYI-2, CaGLYI-7, CaGLYII-6, CaDJ-1A, and CaDLDH-1 under multiple stressors, and downregulation of CaGLYI-3, CaGLYII-1, CaDJ-1B, and CaDJ-1C. Notably, CaGLYI-1 was upregulated under drought and salinity, but downregulated under oxidative, heat, and cold stress [63]. Yu et al. [64] demonstrated that overexpressing ZmGLYI-8 reduced ROS accumulation, detoxified MG and enhanced antioxidant enzyme activity, thereby improving the salt and drought stress resistance of transgenic *Arabidopsis* plants. While our study did not focus on transcript-level analysis, the observed enzyme activity and MG reduction provide functional evidence that supports the role of glyoxalase cycle in stress adaptation. Furthermore, our findings emphasize the potential of melatonin to enhance the efficiency of this pathway, establishing it as a valuable tool for enhancing plant stress tolerance. In support of this, Talaat and Todorova [65] reported that the combined application of melatonin and salicylic acid strengthened both the antioxidant and glyoxalase defence systems in wheat, promoting the detoxification of ROS and MG under salt stress.

In conclusion, this study highlights the significance of both endogenous and exogenous melatonin in enhancing abiotic stress tolerance in pepper plants. Melatonin activates antioxidant systems and key detoxification pathways, such as the glyoxalase cycle, thereby playing a multifaceted role in enhancing stress resilience. The results emphasize that melatonin regulates the GSH/GSSG balance, increases the activity of antioxidant enzymes and promotes detoxification of harmful MG via the glyoxalase pathway. These findings imply that melatonin could be a valuable tool for enhancing drought tolerance, particularly in susceptible genotypes, by supporting vital detoxification processes and mitigating oxidative damage. However, it should be noted that higher levels of endogenous melatonin can determine drought tolerance independently of exogenous melatonin. This makes it worthwhile to develop genotypes rich in this indoleamine for cultivation. Nevertheless, future research focusing on the molecular mechanisms involved could provide more in-depth insights into the potential of melatonin as a biotechnological tool for enhancing plant resistance to environmental stresses.

## 4. Materials and Methods

### 4.1. Material

In our previous research, we evaluated 60 long-fruited pepper genotypes for their endogenous melatonin content and drought stress tolerance. We established a positive relation between pepper endogenous melatonin content and drought tolerance. Based on their endogenous melatonin content, we identified genotypes that were sensitive or tolerant to drought [39]. In the current study, the cultivars “Tuğra” from the Anamas Company (Antalya, Turkey) and “Şahnalı Acı Kıl” from the Altın Company (İzmir, Turkey), which were identified as drought-tolerant and drought-sensitive, respectively, based on the results of the previous study, were used as the research material.

### 4.2. Treatments and Drought Stress Imposition

Seeds of *C. annuum* were sown in small pots (75 cm^3^) containing a peat–perlite mixture (75:25, *v*/*v*). Germination and seedling growth took place in a controlled-environment chamber (25/20 ± 1 °C, day/night) under a 16 h photoperiod, 150 µmol m^−2^ s^−1^ light intensity, and 65 ± 5% relative humidity. Environmental parameters were checked daily, and minor adjustments were made when necessary to keep the growth conditions stable.

Once the seedlings produced four true leaves, melatonin treatment began. Fifteen mL of melatonin solution (0 or 5 µM) was gently applied to the soil around each plant so that the solution could penetrate the root zone evenly. The 5 µM concentration was selected based on our earlier findings in pepper seedlings [39].

To induce drought stress, irrigation was stopped 24 h after melatonin application and withheld for 12 consecutive days. Preliminary trials had shown that this period was sufficient to reduce the leaf water potential to roughly −1.5 MPa, indicating a reliable drought condition.

Four experimental groups were used: C (control), CM (melatonin-control), D (drought), and DM (melatonin-drought). The design followed a randomized complete block pattern with four replicates, each consisting of 30 plants (*n* = 480). At the end of the stress period, all plants were harvested at once for morphological, physiological, and biochemical evaluations.

### 4.3. Determination of Leaf Melatonin, Chlorophyll, Proline Content, as Well as Water Potential

The leaf melatonin was quantified using the HPLC–fluorescence method described by Köklü Ardıç et al. [39], with minor modifications. All operations were performed under dim light to prevent photodegradation. Fresh leaves (0.25 g) were taken from the most recently fully expanded ones, four biological replicates per treatment, and processed immediately.

Tissues were ground in a porcelain mortar with a pestle and transferred into heat-resistant tubes (12 × 75 mm) containing 3 mL chloroform. The tubes were kept at 4 °C in the dark and shaken gently (150 rpm) for 17 h. After centrifugation at 6000× *g* for 20 min, the supernatant was collected, and the pellet was rinsed with 0.5 mL chloroform. The combined extracts were concentrated under vacuum at 35 °C for about an hour (Centrivap (Labconco Corporation, Kansas City, MO, USA)), sealed, and stored at −20 °C until analysis.

Before HPLC analysis, the dried residues were dissolved in 0.5 mL methanol, vortexed, filtered through a 0.45 µm syringe filter, and transferred to autosampler vials. A Shimadzu Prominence UFLC system equipped with an ODS-2 C18 column (250 × 4.6 mm) and a fluorescence detector was used. The mobile phase contained methanol and 0.1 mM Na_2_HPO_4_/H_3_PO_4_ buffer (40:60, *v*/*v*; pH 4.5) at a flow rate of 0.8 mL min^−1^, with the column kept at 35 °C. Fluorescence was detected at λ_ex = 280 nm and λ_em = 350 nm. Peaks were identified by retention time (~15.6 min) against standards, and concentrations were calculated from an external calibration curve.

A 1000 ppm melatonin stock (Sigma-Aldrich, St. Louis, MO, USA) was serially diluted to produce standards of 1–1000 ppb. Standards and samples were run in duplicate, and melatonin contents were expressed as ng g^−1^ fresh weight.

Chlorophyll determination followed Güneş et al. [66]. Fresh leaves (0.25 g) were placed in 15 mL Falcon tubes with 8 mL of cold 100% acetone (HPLC grade) and ground in a chilled glass mortar until uniform. Extracts were filtered (Whatman No. 42), and absorbance was read at 645 and 663 nm on a UV–Vis spectrophotometer (Shimadzu UV-1800, Kyoto, Japan).Pure acetone served as the blank. Chlorophyll *a*, *b*, and total chlorophyll were computed using standard equations. All extractions were performed quickly and on ice to preserve pigment stability. Chlorophyll contents were expressed as mg kg^−1^ fresh weight (FW).

Proline was analyzed as described by Bates et al. [67]. Fresh tissue (0.25 g) was homogenized in 5 mL of 3% (*w*/*v*) sulfosalicylic acid using a pre-chilled mortar and pestle. The homogenate was centrifuged at 6000× *g* for 5 min (25 °C), and 1 mL of the supernatant was mixed with 1 mL acid-ninhydrin and 1 mL glacial acetic acid. The mixture was heated at 100 °C for 1 h, cooled immediately in ice, and 4 mL toluene was added. After vigorous vortexing, the toluene layer was separated, and its absorbance was measured at 520 nm. Proline concentration was determined from a standard curve and expressed as mM kg^−1^ FW.

### 4.4. Determination of Leaf H_2_O_2_ Content, Tissue Electrical Conductivity (EC), TBARS Level and Visual Damage Index

H_2_O_2_ content was determined according to the method of Özden et al. [68]. Fresh leaf tissue (0.25 g) from randomly selected plants in each replication was homogenized in 3 mL of ice-cold 0.1% (*w*/*v*) trichloroacetic acid (TCA) using a pre-chilled porcelain mortar and pestle. The homogenate was transferred into 2 mL microcentrifuge tubes and centrifuged at 10,000× *g* for 10 min at 4 °C.

A 100 µL aliquot of the resulting supernatant was mixed with 100 µL of potassium phosphate (K–P) buffer and 1.5 mL of potassium iodide (KI) in 5 mL reaction tubes. After briefly vortexing, absorbance was recorded at λ = 390 nm using a UV–Vis spectrophotometer. The blank contained deionized water instead of the sample extract but was treated identically. Hydrogen peroxide concentration was calculated from a standard curve (1–500 nmol H_2_O_2_) and expressed as µM kg^−1^ fresh weight (FW).

Membrane integrity was evaluated by measuring leaf tissue electrical conductivity (EC) according to Korkmaz et al. [69]. Leaf discs (1 cm diameter) were excised from two randomly selected surviving seedlings per replicate. The discs were placed in 50 mL Falcon tubes containing 20 mL of deionized water and shaken gently for 24 h. The initial electrical conductivity of the bathing solution was recorded as EC_1_ (µS cm^−1^). The samples were then autoclaved at 121 °C for 15 min to completely disrupt cell membranes, cooled to room temperature (≈25 °C), and the final conductivity (EC_2_ µS cm^−1^) was measured. Membrane damage (%) was calculated as (EC_1_/EC_2_) × 100.

Lipid peroxidation, expressed as thiobarbituric acid reactive substances (TBARS), was determined following the procedure of Zhang et al. [70]. Fresh leaves (0.25 g per replicate) were ground on ice in a chilled mortar with 6 mL of 1% (*w*/*v*) TCA until a uniform slurry was obtained. The homogenate was centrifuged at 6000× *g* for 5 min at 25 °C to obtain a clear supernatant.

An aliquot of the supernatant (1 mL) was mixed with 4 mL of 0.6% (*w*/*v*) thiobarbituric acid (TBA) prepared in 20% (*w*/*v*) TCA. The mixture was heated in a water bath at 100 °C for 20 min and immediately cooled in an ice bath to stop the reaction. After cooling, samples were centrifuged again at 6000× *g* for 5 min at 25 °C to remove turbidity. Absorbance values of the supernatant were measured at λ = 450, 532, and 600 nm using a UV–Vis spectrophotometer. A reagent blank was prepared in parallel by substituting deionized water for the sample extract. TBARS concentration was calculated as mM kg^−1^ FW.

**Figure 6:**
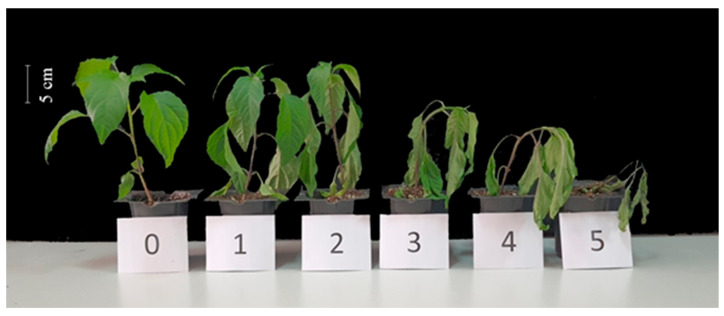
Visual assessment of drought stress severity in pepper plants. 0—none; 1—slight; 2—moderate; 3—severe; 4—extensive; and 5—dead. The image was taken from Köklü Ardıç et al. [39].

The extent of visible injury was evaluated using a 0–5 visual scale described by Köklü Ardıç et al. [39]: 0 = no visible injury, 1 = slight injury, 2 = moderate injury, 3 = severe injury, 4 = extensive injury, and 5 = dead plant (Figure 6). Mean visual damage indices for each treatment were calculated by assigning numerical values (0–5) to individual scores and averaging them across plants.

### 4.5. Determination of Enzyme Activities

For catalase (CAT, EC 1.11.1.6) analysis, fresh leaf tissue (0.25 g) from each genotype and replicate was homogenized on ice in pre-chilled porcelain mortars using an extraction buffer containing 50 mM Tris–HCl (Trisma base), 0.1 mM EDTA, 1 mM PMSF, and 2 mM DTT. The homogenate was centrifuged at 14,000× *g* for 30 min at 4 °C, and the resulting supernatant, containing soluble proteins, was stored at −20 °C until analysis. Total soluble protein content was quantified according to the Bradford [71].

Catalase activity was assayed following Bergmeyer [72], based on the decline in absorbance accompanying H_2_O_2_ decomposition at λ = 240 nm. The reaction mixture (final volume = 1 mL) contained 920 µL of 50 mM sodium-phosphate buffer (pH 7.0) with 0.1 mM EDTA, 70 µL enzyme extract, and 10 µL of 3% (*v*/*v*) H_2_O_2_. The enzyme extract was added last. Absorbance changes were recorded every 5 s for 70 s in kinetic mode, with a reagent blank prepared using deionized water instead of enzyme extract. CAT activity, expressed as the rate of H_2_O_2_ decomposition, was calculated using the molar extinction coefficient *ε* = 39.4 mM^−1^ cm^−1^.

SOD (EC 1.15.1.1) extraction followed the procedure of Köklü Ardıç et al. [39]. Fresh leaf tissue (0.25 g) was homogenized in 3 mL of ice-cold 0.1 M potassium-phosphate buffer (pH 7.5) containing 10 mM KCl, 2.5 mM DTT, 1 mM EDTA, 1.25 mM PEG-4000, and 1 mM PMSF (dissolved in ethanol). The homogenate was centrifuged at 15,000× *g* for 20 min at 4 °C. The supernatant was sequentially filtered through two layers of Miracloth and one layer of filter paper, then desalted on a PD-10 column to obtain the crude soluble protein fraction. Protein content was determined by the Bradford method [71], and extracts were stored at −20 °C.

Before measurement, all reagents were equilibrated to 25 °C. Each replicate was run in two sets (light and dark), and all reactions were performed in duplicate. For the light reactions (final volume = 3.0 mL), 1.15 mL of 0.1 M phosphate buffer (pH 8.0), 500 µL riboflavin, 500 µL 150 mM methionine, 250 µL 1.2 mM EDTA, 500 µL NBT (840 µM), and 100 µL enzyme extract were added into 15 mL glass tubes. Then, the tubes were illuminated under four cold white fluorescent OSRAM lamps (4 × 23 W; ~1500 lm each), while dark controls were kept at 25 °C in complete darkness. After 1 h, absorbance was recorded at λ = 560 nm. One unit (U) of SOD activity was defined as the amount of enzyme required to inhibit NBT photoreduction by 50%.

POX (EC 1.11.1.7) extraction followed the same homogenization steps used for CAT. Fresh leaf tissue (0.25 g) was ground in ice-cold extraction buffer, and the resulting supernatant served as the crude enzyme extract. Total soluble protein was measured using the Bradford method [71].

POX activity was measured as described by Dolatabadian et al. [73] by monitoring guaiacol oxidation in the presence of H_2_O_2_. Two stock solutions were first prepared: (1) 2% (*w*/*v*) gelatin + 0.1 mM DAB (3,3′-diaminobenzidine) and (2) 0.05 M citric acid + 0.08 M sodium-phosphate buffer (pH 7.0). Equal volumes of both solutions were mixed to obtain the guaiacol reaction mixture.

For each assay, 920 µL reaction mixture, 15 µL 30% H_2_O_2_, and 65 µL enzyme extract were added into a 1 mL quartz cuvette. The increase in absorbance was recorded at λ = 465 nm every 5 s for 70 s in kinetic mode. A blank cuvette containing deionized water instead of enzyme extract was run in parallel. POX activity was calculated from the linear increase in absorbance at λ = 465 nm using the extinction coefficient for tetraguaiacol: *ε* = 26.6 mM^−1^ cm^−1^.

GST (EC 2.5.1.18) activity was determined according to the modified method of Nagalakshmi and Prasad [74]. Assays were performed at 30 °C. The reaction mixture (1 mL final volume) contained 0.1 M potassium-phosphate buffer (pH 8.0) with 1 mM EDTA, 10 mM reduced glutathione (GSH), and 1 mM 1-chloro-2,4-dinitrobenzene (CDNB). GSH stock was prepared in phosphate buffer and maintained at 4 °C along with the enzyme extracts. CDNB was dissolved in ethanol (1.8 mL per 3 mL stock) and brought to final volume with buffer.

For each reaction, 795 µL phosphate buffer (with EDTA), 100 µL GSH, 5 µL CDNB, and 100 µL enzyme extract were combined sequentially in a 1 mL quartz cuvette. Absorbance at λ = 340 nm was recorded for 5 min. A blank was prepared using deionized water in place of enzyme extract. Because non-enzymatic conjugation of GSH with CDNB occurs spontaneously, a pre-incubation control (without enzyme) was included and allowed to stand for ~5 min before enzyme addition to start the reaction.

GR (EC 1.6.4.2) activity was measured using a modified method of Esterbauer and Grill [75]. All assay components were equilibrated to 30 °C prior to use. The reaction mixture contained 200 µL of 0.1 M potassium-phosphate buffer (pH 7.5) with 1 mM EDTA, 50 µL of 50 mM MgCl_2_ (prepared without EDTA), 25 µL of 8 mM NADPH, and 25 µL of 0.16 M oxidized glutathione (GSSG). The final reaction volume was 700 µL. The reaction was started by adding 100 µL enzyme extract, and the decrease in absorbance at λ = 340 nm was monitored for 5 min in kinetic mode. A blank without enzyme was run simultaneously. GR activity was calculated using the molar extinction coefficient for NADPH: *ε* = 6.22 mM^−1^ cm^−1^.

GPX (EC 1.11.1.9) activity was assayed according to the NADPH-dependent coupled method of Esterbauer and Grill [75], with reagents equilibrated to 30 °C. The reaction is based on the H_2_O_2_-dependent oxidation of GSH by GPX, producing GSSG, which is subsequently reduced by GR and NADPH. The decrease in NADPH absorbance at λ = 340 nm reflects GPX activity.

Each 1 mL reaction cuvette contained: 175 µL 0.1 M potassium-phosphate buffer (pH 8.0); 50 µL 10 mM EDTA; 50 µL 1 M NaCl; 50 µL 10 mM GSH, 15 µL 8 mM NADPH, 50 µL 30% H_2_O_2_ and 10 µL GR solution (100 U per 0.5 mL stock)

Reactions were initiated by adding 100 µL enzyme extract, and NADPH oxidation was recorded at λ = 340 nm for 5 min in kinetic mode. A blank cuvette lacking enzyme extract served as the control. GPX activity was calculated using the molar extinction coefficient for NADPH: ε = 6.22 mM^−1^ cm^−1^.

### 4.6. Determination of Glutathione Levels

Sahoo et al. [76] outlined the method used to extract glutathione from plant tissues. Leaf tissue (1.0 g) was homogenized in 10 mL of 6% metaphosphoric acid containing 1 mM EDTA at 4 °C. The homogenate was centrifuged at 12,000× *g* for 15 min at 4 °C, and the supernatant was stored at 4 °C until analysis.

Total glutathione (GSG) content was determined following a slightly modified version of the method described by Sahoo et al. [76]. In a 15 mL tube, 4 mL of extract was mixed with 1 mL of 0.5 M potassium phosphate buffer (pH 7.5), 100 μL of 10 mM DTNB (prepared in ethanol), and 200 μL of 0.5 mM BSA. The mixture was incubated in a water bath at 37 °C for 20 min, cooled to room temperature, and absorbance was measured at λ = 412 nm. Total GSG concentration was calculated using the extinction coefficient: $\varepsilon =0.017\;{\mathrm{mM}}^{-1}{\mathrm{cm}}^{-1}$.

Reduced glutathione (GSH) content was determined according to Sahoo et al. [76]. Initially, 600 µL of 100 mM potassium phosphate buffer (K–P; pH 7.5) and 40 µL of 0.6 mM DTNB were added to a quartz cuvette and placed in a spectrophotometer. After establishing a baseline absorbance at 412 nm, the reaction was initiated by adding 360 µL of the crude extract. The change in absorbance was recorded every 5 s for 2 min. A standard calibration curve was constructed using GSH solutions ranging from 1 to 700 µM. GSH levels were calculated based on the rate of absorbance change and expressed as nmol g^−1^ fresh weight (FW).

The oxidized glutathione (GSSG) was quantified according to Sahoo et al. [76]. To block reduced glutathione (GSH) and prevent its reformation during analysis, 30 μL of 2-vinylpyridine (diluted 1:10 in phosphate buffer and kept on ice) was added to 1 mL of the crude extract. Samples were incubated at 25 °C for 1 h to ensure complete derivatization of GSH. Following incubation, 100 μL of the treated extract was transferred into a quartz cuvette. The reaction mixture contained 600 μL of 100 mM potassium phosphate buffer (pH 7.5) supplemented with 5 mM EDTA, 100 μL of diluted yeast glutathione reductase (GR; 20 U mL^−1^), and 100 μL of 10 mM DTNB prepared in 100 mM phosphate buffer without EDTA. The cuvette was placed in a spectrophotometer set to λ = 412 nm. The reaction was initiated by adding NADPH (2.5 mM in phosphate buffer) to the cuvette, and the change in absorbance was monitored every 5 s for 2 min. A standard calibration curve was generated using known concentrations of GSSG, and results were expressed as nmol g^−1^ fresh weight (FW). The reaction was monitored at λ = 412 nm and quantified by a standard curve (same protocol, various GSSG concentrations).

### 4.7. Determination of MG Content and Gly I and Gly II Enzyme Activities

MG content was quantified following the method of Hoque et al. [77] with minor modifications. Fresh leaf tissue (0.5 g) was homogenized in 3 mL of 0.5 M perchloric acid and centrifuged at 11,000 rpm (~12,000 × g) for 10 min at 4 °C. The resulting supernatant was transferred to a clean tube containing 10 mg activated charcoal and incubated at room temperature (~25 °C) for 15 min. After incubation, samples were centrifuged again at 11,000 rpm for 10 min. The supernatant was neutralized with a saturated potassium-carbonate solution and centrifuged once more under the same conditions. For derivatization, 250 µL of 7.2 mM 1,2-diaminobenzene, 100 µL of 5 M perchloric acid, and 650 µL of neutralized extract were mixed and incubated for 25 min at room temperature. Absorbance was recorded at λ = 335 nm using a UV–Vis spectrophotometer. Methylglyoxal concentration was calculated from a standard calibration curve and expressed as µmol g^−1^ fresh weight (FW).

For Gly I (EC 4.4.1.5) and Gly II (EC 3.1.2.6) analyses, a slightly modified version of the method described by Hasanuzzaman and Fujita [60] was used. Leaf tissue (0.1 g) was homogenized in 1 mL of ice-cold extraction solution containing 50 mM potassium-phosphate buffer (pH 7.5), 100 mM KCl, 1 mM ascorbate, 5 mM β-mercaptoethanol, and 10% (*w*/*v*) glycerol. The homogenate was centrifuged at 13,000× *g* for 20 min at 4 °C, and the resulting supernatant was immediately used for enzyme assays. All procedures were carried out at 0–4 °C.

To determine Gly I activity, reactions were carried out in 1.0 mL quartz cuvettes containing 780 µL of 100 mM K-P buffer (pH 7.5), 174 µL of 15 mM MgSO_4_, 8.5 µL of 1.7 mM reduced glutathione (GSH), and 17.5 µL of 3.5 mM methylglyoxal (MG). The reaction was initiated by adding 20 µL of enzyme extract, and the increase in absorbance was monitored at λ = 240 nm every 5 s for 1 min in kinetic mode. Gly I activity was calculated using the extinction coefficient *ε* = 3.37 mM^−1^ cm^−1^ (for S-D-lactoylglutathione at 240 nm).

Gly II activity was assayed by monitoring the formation of reduced glutathione (GSH) at λ = 412 nm using DTNB. The 1.0 mL reaction mixture consisted of 860 µL of 100 mM Tris–HCl buffer (pH 7.2), 66.6 µL of 0.2 mM DTNB (dissolved in ethanol), and 33.4 µL of 1 mM S-D-lactoylglutathione. The reaction was initiated by adding 40 µL of enzyme extract, and absorbance was recorded every 5 s for 1 min in kinetic mode. A reagent blank without enzyme extract was run in parallel. Gly II activity was calculated using the molar extinction coefficient for TNB (5-thio-2-nitrobenzoic acid): *ε*_412_ = 13.6 mM^−1^ cm^−1^.

### 4.8. Evaluation of Results

Data were analyzed using the JMP software package (Version 16). The Shapiro–Wilk test was employed to verify normality, and O’Brien’s test was applied to evaluate homogeneity of variances. Both assumptions were satisfied (*p* > 0.05 for all variables). Therefore, significance was assessed at α = 0.05 using one-way analysis of variance (ANOVA) performed separately for each cultivar. Treatment means were compared using Tukey’s multiple comparison test.

## 5. Conclusions

This study clearly demonstrated the critical role of melatonin in enhancing drought tolerance in *C. annuum* L., both when accumulated naturally and when applied externally. Melatonin improved photosynthetic pigment content delayed senescence and maintained tissue water balance under drought stress. It also stimulated proline accumulation to strengthen osmotic adjustment.

Melatonin treatment reinforced the antioxidant defence system by increasing the activities of SOD, CAT and POX, thereby reducing the accumulation of H_2_O_2_, lipid peroxidation and membrane damage. Furthermore, melatonin modulated glutathione metabolism and activated the glyoxalase pathway. This resulted in higher GSH/GSSG ratios and decreased MG levels. Together, these effects mitigate oxidative and carbonyl stresses.

**Figure 7:**
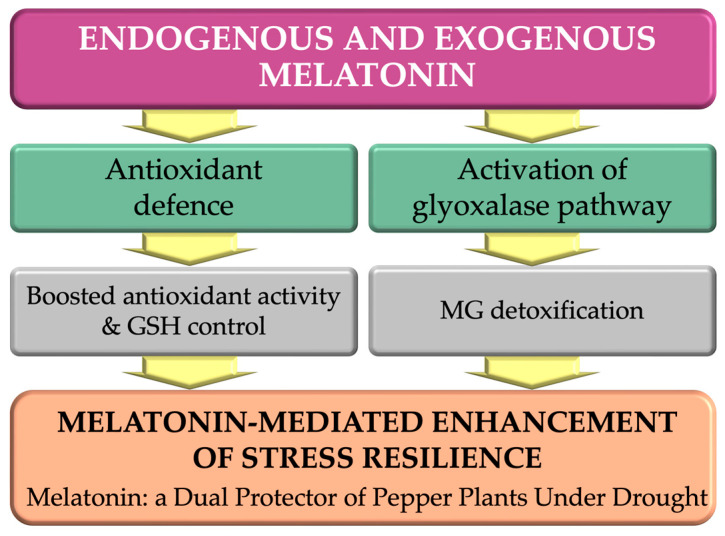
Melatonin enhances drought tolerance in pepper plants via two complementary mechanisms: activation of antioxidant defence and glyoxalase-mediated detoxification. Both endogenous and exogenous melatonin contribute to stress resilience by reducing oxidative damage and promoting cellular detoxification.

These findings reveal a dual mechanism through which melatonin enhances plant resilience by synchronizing the antioxidant defence system with methylglyoxal detoxification (Figure 7). The higher endogenous melatonin content in drought-tolerant genotypes suggests that endogenous melatonin biosynthesis underlies natural stress tolerance. Conversely, exogenous melatonin supplementation can partially compensate for this deficit in susceptible plants.

Overall, this work provides new insight into the multifaceted protective role of melatonin, supporting its application as a promising biotechnological tool for improving crop tolerance to drought and other abiotic stresses.

## Figures and Tables

**Table 1:** The effects of melatonin application on parameters related to plant viability under drought conditions: endogenous melatonin content, chlorophyll and proline levels, as well as water potential in pepper varieties with different drought stress tolerance.

Genotype	Treatments	Melatonin (µg kg^−1^ FW)	Chlorophyll (mg kg^−1^ FW)	Proline (mM kg^−1^ FW)	Water Potential (−MPa)
Drought Sensitive	C	23.9 ± 1.82 ^d^	39.9 ± 0.28 ^b^	24.2 ± 0.32 ^d^	1.25 ± 0.01 ^c^
	CM	61.6 ± 3.83 ^c^	56.5 ± 0.11 ^a^	62.0 ± 0.17 ^c^	0.90 ± 0.01 ^d^
	D	97.5 ± 3.80 ^b^	28.7 ± 0.40 ^c^	76.3 ± 0.43 ^b^	1.74 ± 0.01 ^a^
	DM	120.4 ± 6.80 ^a^	40.4 ± 0.47 ^b^	100.8 ± 0.50 ^a^	1.39 ± 0.01 ^b^
	ANOVA	***	***	***	***
Drought Tolerant	C	111.3 ± 1.30 ^c^	71.8 ± 0.32 ^b^	52.2 ± 0.30 ^d^	1.04 ± 0.01 ^c^
	CM	153.4 ± 1.60 ^b^	80.3 ± 0.58 ^a^	72.1 ± 0.48 ^c^	0.93 ± 0.01 ^d^
	D	207.6 ± 15 ^a^	58.2 ± 0.22 ^d^	105.9 ± 0.30 ^b^	1.21 ± 0.01 ^a^
	DM	245.6 ± 4.42 ^a^	63.8 ± 0.70 ^c^	135.0 ± 0.76 ^a^	1.10 ± 0.01 ^b^
	ANOVA	***	***	***	***

C: control-no stress; CM: control plant pre-treated with 5 µM melatonin; D: drought stress; and DM: drought-stressed plants pre-treated with 5 µM melatonin. Different letters in the same column indicate statistically significant differences. *** *p* < 0.001. Values are given as mean ± standard error.

**Table 2:** Effects of melatonin application on parameters related to oxidative damage: H_2_O_2_ content, electrical conductivity (EC), TBARS level and visual damage index in pepper seedlings with different drought stress tolerance.

Genotype	Treatments	H_2_O_2_ (µM kg^−1^ FW)	EC (%)	TBARS (mM kg^−1^ FW)	Injury Index (0–5 Point)
Drought Sensitive	C	1.20 ± 0.01 ^c^	75.0 ± 1.11 ^c^	8.7 ± 0.32 ^b,c^	1.25 ± 0.03 ^b^
	CM	1.00 ± 0.02 ^d^	68.2 ± 0.17 ^d^	7.0 ± 0.17 ^c^	1.19 ± 0.01 ^b^
	D	1.77 ± 0.01 ^a^	88.6 ± 0.71 ^a^	12.4 ± 0.43 ^a^	4.10 ± 0.04 ^a^
	DM	1.45 ± 0.01 ^b^	80.1 ± 0.51 ^b^	10.1 ± 0.50 ^b^	4.00 ± 0.03 ^a^
	ANOVA	***	***	***	***
Drought Tolerant	C	0.86 ± 0.02 ^b,c^	11.6 ± 1.01 ^a,b^	5.2 ± 0.37	1.24 ± 0.01 ^c^
	CM	0.78 ± 0.0324 ^c^	10.5 ± 1.85 ^b^	4.7 ± 0.63	1.19 ± 0.03 ^c^
	D	1.00 ± 0.03 ^a^	17.8 ± 1.39 ^a^	6.5 ± 0.29	3.57 ± 0.01 ^a^
	DM	0.91 ± 0.01 ^a,b^	12.2 ± 0.79 ^a,b^	5.5 ± 0.79	3.10 ± 0.04 ^b^
	ANOVA	***	*	NS	***

C: control-no stress; CM: control plant pre-treated with 5 µM melatonin; D: drought stress; and DM: drought-stressed plants pre-treated with 5 µM melatonin. Different letters in the same column indicate statistically significant differences. * *p* < 0.05, *** *p* < 0.001; NS: not statistically significant. Values are given as mean ± standard error.

**Table 3:** Content of total glutathione (GSG), reduced glutathione (GSH), oxidized glutathione (GSSG), and GSH/GSSG ratio in pepper seedling of drought-tolerant and -sensitive genotypes after treatment with melatonin.

Genotype	Treatments	GSG (nM g^−1^ FW)	GSH (nM g^−1^ FW)	GSSG (nM g^−1^ FW)	GSH/GSSG Ratio
Drought Sensitive	C	215.4 ± 1.8 ^d^	20.3 ± 1.1 ^d^	195.1 ± 1.32 ^a^	0.10 ± 0.01 ^d^
	CM	244.9 ± 3.8 ^c^	77.3 ± 4.8 ^c^	167.6 ± 1.77 ^b^	0.46 ± 0.03 ^c^
	D	374.2 ± 3.8 ^b^	254.1 ± 3.2 ^b^	120.1 ± 1.21 ^c^	2.12 ± 0.03 ^b^
	DM	475.5 ± 6.8 ^a^	376.1 ± 5.3 ^a^	99.4 ± 2.51 ^d^	3.79 ± 0.09 ^a^
	ANOVA	***	***	***	***
Drought Tolerant	C	413.8 ± 1.3 ^d^	285.4 ± 0.6 ^d^	128.4 ± 1.27 ^a^	2.22 ± 0.02 ^d^
	CM	524.3 ± 1.6 ^c^	417.5 ± 1.0 ^c^	106.8 ± 1.83 ^b^	3.91 ± 0.07 ^c^
	D	586.3 ± 15 ^b^	502.2 ± 4.3 ^b^	84.1 ± 1.41 ^c^	5.97 ± 0.16 ^b^
	DM	664.6 ± 4.4 ^a^	609.1 ± 4.2 ^a^	55.4 ± 3.01 ^d^	11.12 ± 0.63 ^a^
	ANOVA	***	***	***	***

C: control-no stress; CM: control plant pretreated with 5 µM melatonin; D: drought stress; and DM: drought-stressed plants pretreated with 5 µM melatonin. Different letters in the same column indicate statistically significant differences. *** *p* < 0.001. Values are given as mean ± standard error.

## Data Availability

The data supporting the findings of this study were generated in the laboratory but are not publicly available due to privacy and ethical restrictions.

## Abbreviations

The following abbreviations are used in this manuscript:

APX	Ascorbate Peroxidase
CAT	Catalase
D	Drought Stress
C	Control (No Stress)
CM	Control + Melatonin
DM	Drought Stress + Melatonin
EC	Electrical Conductivity
FW	Fresh Weight
Gly I	Glyoxalase I
Gly II	Glyoxalase II
GPX	Glutathione Peroxidase
GR	Glutathione Reductase
GSH	Reduced Glutathione
GSSG	Oxidized Glutathione
GSG	Total Glutathione
GST	Glutathione S-transferase
H_2_O_2_	Hydrogen Peroxide
MG	Methylglyoxal
POX	Peroxidase
ROS	Reactive Oxygen Species
SOD	Superoxide Dismutase
TBARS	Thiobarbituric Acid Reactive Substances
Ψw	Water Potential
